# Evaluation of the Health‐Related Quality of Life and Mental Health of Parents With Children and Adolescents With a Rare Disease Based on the Results of a Randomized Controlled Trial to Investigate a Family‐Based Intervention and an Online Intervention for Affected Families (CARE‐FAM‐NET)

**DOI:** 10.1111/famp.70041

**Published:** 2025-05-15

**Authors:** Antonia Steinberg, Johannes Boettcher, Anna Leidger, Ania C. Muntau, Jonas Denecke, Nicole Kaiser, Anne Daubmann, Antonia Zapf, Karl Wegscheider, Ann Kathrin Ozga, Anna Isabella Suling, Monika Bullinger, Julia Quitmann, Jörg Dirmaier, Stefanie Witt, Farhad Rezvani, Christine Mundlos, Lisa Biehl, Miriam Rassenhofer, Jörg M. Fegert, Dunja Tutus, Gerald Willms, Jan Zeidler, Nicolas Pardey, Johann‐Matthias Graf von der Schulenburg, Silke Wiegand‐Grefe

**Affiliations:** ^1^ Department of Psychiatry and Psychotherapy University Medical Center Hamburg‐Eppendorf Hamburg Germany; ^2^ Department of Child and Adolescent Psychiatry, Psychosomatics and Psychotherapy University Medical Center Hamburg‐Eppendorf Hamburg Germany; ^3^ University Children's Hospital University Medical Center Hamburg‐Eppendorf Hamburg Germany; ^4^ German Center for Child and Adolescent Health (DZKJ) Hamburg Germany; ^5^ Institute of Medical Biometry and Epidemiology University Medical Center Hamburg‐Eppendorf Hamburg Germany; ^6^ Institute and Polyclinic for Medical Psychology University Medical Center Hamburg‐Eppendorf Hamburg Germany; ^7^ ACHSE e.V.: Alliance of Chronic Rare Diseases Berlin Germany; ^8^ Child and Adolescent Psychotherapy Ulm University Hospital Ulm Germany; ^9^ Institute for Applied Quality Promotion and Research in Healthcare GmbH aQua Institute Göttingen Germany; ^10^ Center for Health Economics Research Hannover (CHERH) Leibniz Universität Hannover Hanover Germany

**Keywords:** family intervention, health‐related quality of life, mental health, parents, rare disease

## Abstract

Parents caring for children with rare diseases are more impaired regarding health‐related quality of life (HRQoL) and mental health than healthy controls and norm data. To address the research gap in psychological care for these parents, this study evaluates the effectiveness of two family‐based interventions. The children affected by rare disease and their families network (CARE‐FAM‐NET) study is a multicenter randomized controlled 2 × 2 factorial trial for affected families with children (0–21 years). This paper focuses on evaluating the impact of two interventions, one face‐to‐face (CARE‐FAM) and one online (WEP‐CARE), on the HRQoL and mental health of parents. One thousand, one hundred sixty‐eight parents participated: TAU = 291, CARE‐FAM = 296, WEP‐CARE = 300, and CARE‐FAM + WEP‐CARE combined = 281. Data were collected at four time points over a period of 18 months using standardized questionnaires. The results had to be interpreted exploratively. The results indicate that there are no clinically relevant differences in the parents' HRQoL and mental health between the treatment groups. However, time‐dependent differences in the intervention effects for WEP‐CARE were observed. Although the results did not show clear relevant differences between conditions, trends in improvement in HrQoL and mental health were identified. CARE‐FAM shows a greater reduction in parental distress and WEP‐CARE shows a greater distortion of distress, particularly at T3 and T4. Given the exploratory nature of this study, it highlights the urgent need for further confirmatory research in this area.

## 1 Introduction

Many rare diseases (RDs) manifest in childhood and continue throughout life, placing a significant burden on family members, especially parents (Kirby 2012; Zurynski et al. 2008; Baumbusch et al. 2018). Affected parents often bear the primary responsibility for caregiving and provide substantial physical, practical, and emotional support (Boettcher et al. 2020, 2021; Candy et al. 2011). Although there have been increasing efforts in recent years to address the psychosocial burden of parents of children with RD, there is still a lack of data from large multicenter studies.

RDs are defined as affecting no more than 5 in 10,000 people in the EU (Moliner and Waligora 2017). In Germany, it is estimated that between 2.4 and 5 million children and adolescents are affected by more than 8000 different RDs (Mundlos 2017). RDs describe a heterogeneous group of highly complex clinical pictures that are often chronic, mostly occur at an early age, and can be associated with a limited life expectancy (Pelentsov et al. 2016). Caring for people with these conditions requires considerable effort, particularly the involvement of parents (Frank et al. 2014). Parents often experience social isolation, withdrawing from social activities due to caregiving demands and concerns about social stigma (Witt et al. 2023). They often feel uncomfortable when asked about their child's illness in public, leading to a sense of being perceived differently by others. On average, it takes more than 5 years for an individual to receive a correct diagnosis of RD (Vandeborne et al. 2019). Some individuals wait decades for a diagnosis, while others never receive one (Dong et al. 2020).

Research consistently indicates that parents caring for children with RDs have impaired health‐related quality of life (HRQoL; Boettcher et al. 2021), although comparable to parents of children with other chronic diseases (Cohn et al. 2020). In addition, parents of children with RDs often experience poor mental health with high levels of stress, anxiety, and depression as they navigate the complexities of managing their child's condition (Rihm et al. 2022). While some parents find positive aspects to their role (McMullan et al. 2020), the burden is compounded when the child's condition remains undiagnosed, exacerbating symptoms of depression and anxiety in parents (McConkie‐Rosell et al. 2018). Access to support services, knowledgeable professionals, and advocacy groups is often limited, further complicating the care of children with RD (Boettcher et al. 2021). Recognizing the profound impact on parents' HRQoL and mental health is crucial for healthcare professionals (Boettcher et al. 2021; Fuerboeter et al. 2021). Particularly in this area, the psychosocial care of families with a child with an RD, there is a lack of research and studies on the effectiveness and usefulness of interventions (Lyon et al. 2024).

Although previous research has called for psychological support for affected families (Boettcher et al. 2021; Frank et al. 2014; Fuerboeter et al. 2021), family‐oriented programs targeting parental HRQoL and mental health are still lacking. To adequately address such specialized services, the children affected by rare disease and their families network (CARE‐FAM‐NET) study, on which this review is based, investigated the effectiveness of family‐based interventions for these families (Boettcher et al. 2020). With the CARE‐FAM‐NET study, we aim to fill the gap in psychosocial care in this area with two new interventions: the online intervention WEP‐CARE, which is particularly helpful for parents who already have difficulty coping with all the tests and are therefore already very stressed, and a face‐to‐face intervention CARE‐FAM, which has the advantage of a therapeutic effect. The relationship between therapist and patientcan also have a major impact, unlike the online intervention. Therefore, in this study, first, all parents (both symptomatic (distressed) and non‐symptomatic) in the intervention groups will have higher HRQoL 6 months after randomization compared to parents who did not receive the intervention. Second, all parents (both symptomatic and non‐symptomatic) in the intervention groups will have fewer psychological symptoms and better mental health 6 months after randomization than parents who did not receive the intervention. The group differences remain stable at further measures at 12 and 18 months.

## 2 Method

The data were collected as part of the CARE‐FAM‐NET study, which is being carried out by a research network coordinated by the Department of Child and Adolescent Psychiatry and Psychotherapy, Psychotherapy and Psychosomatics at the University Medical Center Hamburg‐Eppendorf in cooperation with 40 other partners. The study was funded by the Innovation Committee of the Federal Joint Committee (G‐BA, project number: 01NVF17028). The trial was pre‐registered in the German Clinical Trials Register (DRKS00015859) and ClinicalTrials.gov (NCT04339465). Ethical approval was granted by the Ethics Committee of the Regional Medical Association of Hamburg (PV5749). The methods used in this study comply with the tenets of the Declaration of Helsinki. Consent to participate was obtained from all participants included in the study. Parents signed informed consent, and children were allowed to fill in questionnaires for themselves from age 10. Every individual participating in the study was informed that they could withdraw from the study at any time and without any consequences.

### 2.1 Study Design

The family interventions CARE‐FAM and WEP‐CARE for families with children with RD were implemented in each of the 17 participating centers, which were also involved in the design of the CARE‐FAM‐NET program, and evaluated in a 2 × 2 factorial randomized controlled trial (RCT). The total sample of the CARE‐FAM‐NET study consists of 687 families. These families were assigned to one of four groups: CARE‐FAM (CF), WEP‐CARE (WC), CARE‐FAM + WEP‐CARE (CF + WC), or treatment as usual (TAU). CF is a face‐to‐face intervention, a semi‐structured program targeting mental health problems in children with RD, their parents and siblings, developed to apply a low‐frequency, family‐oriented intervention based on a developmental model (Mattejat et al. 2000) and the family counseling approach for children with mentally ill parents (Wiegand‐Grefe et al. 2011). The CF intervention took place over 6 months, with sessions taking place every 2–3 weeks. Each family attended several sessions, including an initial session, two sessions for the parents, one for the child, and three for the whole family. The interventions were delivered by qualified therapists with psychotherapeutic training, following specialized training and regular supervision, which was mandatory. Implementation followed contractual guidelines. Nationwide, 34 therapists delivered the CARE‐FAM intervention in 17 centers (for further details of the intervention process see Boettcher et al. 2020). The CF program was adapted for online and telephone sessions during the COVID‐19 pandemic. The WC intervention is a cognitive‐behavioral intervention delivered via online platforms and was originally developed to support parents of chronically ill children (Fidika et al. 2015). Over 12 to 14 weeks, trained therapists guided one parent in particular from assigned families through a series of 12 structured writing tasks. In WC, designed for anxiety management, the first six writing tasks were considered the ‘minimum therapeutic dose’. Given the asynchronous communication with the therapists, participants had the flexibility to complete the tasks at their own pace, with a recommended rate of one task per week (approximately 45 min). After each submission, personalized feedback was provided within two working days, together with further instructions if necessary. The therapeutic workload varied according to the expertise of the therapist and the complexity of the texts, but was generally comparable to that required by parents. The therapy was delivered by highly qualified therapists with psychotherapeutic training who had undergone specialized training and ongoing supervision. Their training included a 2‐day workshop followed by five supervised writing therapies. Supervision, which was mandatory on a monthly basis, was provided in written form as well as via telephone or web conferencing. Supervisors were required to hold a PP/KJP license and have experience with at least 10 WEP‐CARE therapies. Further details of the intervention process can be found in Boettcher et al. (2020).

For the third intervention, families were assigned to a combination of the CARE‐FAM and WEP‐CARE interventions. TAU represents the standard of care typically provided in regular health care settings without any additional intervention from this trial.

Baseline questionnaires were completed by all groups, followed by a clinical interview. Parents, affected children, siblings and therapists were assessed using different questionnaires. The data were collected and the results were summarized by blind external reviewers. Data were collected at four time points: baseline, and 6, 12, and 18 months after baseline. A sample size calculation for the CARE‐FAM‐NET study was performed for the primary endpoint ‘mental health diagnosis of initially distressed parents’ 18 months after randomization. More information can be found in Boettcher et al. (2021). Note that this sample size calculation was not performed for the endpoints of interest in this study, as these were only secondary endpoints of the CARE‐FAM‐NET study; therefore, the analysis presented is exploratory.

### 2.2 Measures

#### 2.2.1 Sociodemographic and Clinical Variables

In this study, we are only interested in the parents' perspective, so the following parental characteristics are of interest: age, gender, number of children, education and employment status, medical history, and current treatments using a specially designed questionnaire at baseline.

#### 2.2.2 Health‐Related Quality of Life (HRQoL)

The Short Form‐12 (SF‐12; Ware et al. 1996) was used to assess parental HRQoL. The instrument is a 12‐item self‐report questionnaire that assesses both psychological and physical HRQoL. Higher scores indicate better HRQoL. The instrument has shown good cross‐cultural stability and good psychometric properties (Morfeld et al. 2011). The internal consistency of the SF‐12 scale was acceptable (T1: *α* = 0.85, T2: *α* =0.86, T3: *α* = 0.87, T4: *α* =0.87). The Ulm Quality of Life Inventory for Parents of Chronically Ill Children (ULQIE; Goldbeck and Storck 2002) was also used to assess parental HRQoL. The ULQIE measures different facets of HRQoL in five domains with 29 items, with higher scores indicating better HRQoL for parents, particularly in coping with the challenges of their child's illness. Reliability and validity have been shown to be acceptable in a cohort of parents of children with chronic illness (Goldbeck and Storck 2002). The observed internal consistency for the ULQIE scale was poor (T1: *α* = 0.01, T2: *α* = 0.13, T3: *α* = 0.18, T4: *α* = 0.08).

#### 2.2.3 Mental Health

The Brief Symptom Inventory (BSI; Derogatis and Franke 2000) was used to assess parental mental health. The 53‐item BSI captures different aspects of psychological symptoms, providing a nuanced understanding of parents' mental health from multiple dimensions. The BSI evaluates nine distinct symptom dimensions: somatization, compulsivity, interpersonal sensitivity, depression, anxiety, hostility, phobic anxiety, paranoid ideas, and psychoticism, hence providing a comprehensive overview of the parents' emotional and psychological states. In addition to these individual dimensions, the BSI includes three global indices to measure overall psychological symptoms, including the global severity index (GSI). Furthermore, the BSI computes the total score of the three indices (the sum of the sum), consolidating the overall impact and severity of psychological burdens experienced by the parents. Both mean and sum versions of the nine symptom dimensions are used to analyze the data, providing a more comprehensive understanding. Elevated scores on the BSI indicate increased levels of symptoms experienced by parents. Reliability and validity have been shown to be acceptable (Derogatis and Franke 2000). The internal consistency of the BSI total score was excellent (T1: *α* = 0.95, T2: *α* = 0.96, T3: *α* = 0.95; T4: *α* = 0.96).

The Patient Health Questionnaire‐9 (PHQ‐9; Löwe et al. 2002) was also used to assess parental depressive symptoms. The PHQ‐9 has a sensitivity of 78% for diagnosing all depressive disorders in psychosomatic patients and 75% in medical patients. The specificity is 71% and 90%, respectively (Gräfe et al. 2004; Kroenke et al. 2001). The internal consistency of the PHQ‐9 scale was acceptable (T1: *α* = 0.84, T2: *α* = 0.84; T3: *α* = 0.85; T4: *α* = 0.84).

### 2.3 Statistical Analyses

#### 2.3.1 Randomization, Blinding and Recruitment

The Institute for Medical Biometry and Epidemiology of the University Medical Center Hamburg‐Eppendorf (UKE) generated computerized lists for family‐wise randomization with variable block lengths, stratified by recruitment center. According to a 2 × 2 factorial design, each family was randomly assigned to one of four combinations representing the two interventions (CARE‐FAM only, WEP‐CARE only, a combination of CARE‐FAM and WEP‐CARE, or TAU). A central allocation procedure was then carried out by the coordinating study center in Hamburg, which ensured that the allocation was concealed. Families were informed of their group allocation.

### 2.4 Statistical Analysis

Baseline characteristics of the parents were described both overall and separately for the randomized groups. Means and standard deviations were presented for continuous variables and absolute and relative frequencies for categorical variables. The number of missing observations was reported separately for each randomized group. No tests of statistical significance were performed for baseline characteristics; rather, the clinical importance of any imbalance was noted (Senn 1994).

For the outcomes of interest, change from baseline to T2 (at 6 months), T3 (at 12 months) and T4 (at 18 months) was calculated. Linear mixed models were used with differences from baseline as the outcome, recruitment center, family, and family member as nested random effects, and intervention group as fixed effects (two factors: 1st factor CARE‐FAM yes vs. no, 2nd factor WEP‐CARE yes vs. no) and time as a fixed effect and baseline as a covariate. Interactions between time and the intervention group were determined. If the corresponding *p*‐value was > 0.05, they were excluded from the model. Under the assumption of two independent interventions (i.e., the interaction *p*‐value between CARE‐FAM and WEP‐CARE was above 0.05), and in the case of an interaction between time and the intervention group (*p*‐value < 0.05), the contrast between the respective control group and each intervention group was calculated for each time point (T2, T3, T4). If the group‐time interaction *p*‐values were > 0.05, the contrast between the respective control group and each intervention group was calculated over all time points. Adjusted means with 95% confidence interval (CI) and *p*‐values were reported. Intra‐class correlation (ICC) was also reported. For the analysis of the endpoints, the complete analysis set was used, i.e., including parents with baseline measures and values at least at one of the time points T2, T3, and T4. Due to the factorial design, the independence of the two factors was tested by including the interaction between the two factors in the models, with independence indicated by a *p*‐value > 0.05. If the *p*‐value for the interaction was < 0.05 and therefore independence was not present, the estimators, their 95% CIs, and the *p*‐value for each combination of interaction were presented. In the case of interactions of intervention groups with time, this procedure was extended to three‐way interactions, with stepwise backward selection of the interactions. The assumptions regarding the normality of the residuals and the linearity between the independent variables and the dependent variable were examined graphically, i.e., using partial residual plots, residual plots, and quantile‐quantile plots. No interim analysis was performed. Missing values were not imputed for the secondary outcomes analyzed in the paper, and no adjustments were made for multiple testing, i.e., all *p*‐values are descriptive. R version 4.2.3 (package lme4 with function lmer) was used for analysis.

## 3 Results

**FIGURE 1:**
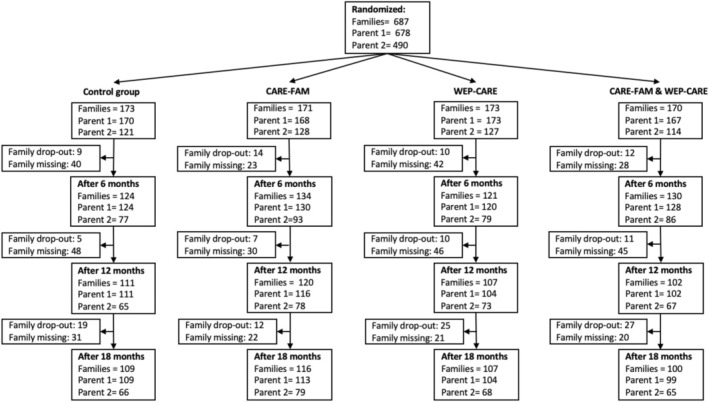
Description of the study population, number of randomized participants, allocation to three intervention groups (CARE‐FAM, WEP‐CARE, CARE‐FAM + WEP‐CARE) and one control group (TAU). Numbers of participants at every measurement point after 6, 12, and 18 m. “Family missing” indicated that the entire family did not provide any information at that time.

Figure 1 provides an overview of the families and parents who participated in the study, illustrating the number of participating parents at each measurement time point. Reasons for drop‐out were not given. Drop‐out was only given family‐wise and not for family members. Some families or family members did not complete the questionnaires but remained in the trial (e.g., a diagnostic interview was conducted), while only those families with no information (no questionnaires and no interviews) were considered drop‐outs. Additionally, some families or family members missed completing questionnaires at specific time points but re‐engaged in the study at later stages. The total number of participating parents was 1168: Control group (TAU, *n* = 291; CARE‐FAM, *n* = 296; WEP‐CARE, *n* = 300; and CARE‐FAM + WEP‐CARE combined, *n* = 281).

### 3.1 Descriptive Statistics

Demographic and baseline characteristics for all four groups are shown in Table 1. No clinically relevant differences between randomized groups were found.

**TABLE 1:** Demographics and baseline characteristics.

	TAU (*N* = 291)	CF (*N* = 296)	WC (*N* = 300)	CF & WC (*N* = 281)	Total (*N* = 1168)
	*M* (SD)	*M* (SD)	*M* (SD)	*M* (SD)	*M* (SD)
Parental age	285 (97.94%)	293 (98.99%)	294 (98.00%)	276 (98.22%)	1148 (98.29%)
	40.99 (7.14)	40.47 (7.59)	41.40 (7.26)	40.40 (6.88)	40.82 (7.23)
	***n* (%)**	***n* (%)**	***n* (%)**	***n* (%)**	***n* (%)**
Female	169/287 (58.9%)	167/295 (56.6%)	171/296 (57.8%)	167/277 (60.3%)	674/1155 (58.4%)
Male	118/287 (41.1%)	128/295 (43.4%)	125/296 (42.2%)	110/277 (39.7%)	481/1155 (41.6%)
Relation to the child with RD					
Mother	166/288 (57.6%)	168/296 (56.8%)	169/297 (56.9%)	165/279 (59.1%)	668/1160 (57.6%)
Father	115/288 (39.9%)	124/296 (41.9%)	119/297 (40.1%)	109/279 (39.1%)	467/1160 (40.3%)
Currently suffering from mental illness	27/285 (9.5%)	33/292 (11.3%)	31/292 (10.6%)	37/272 (13.6%)	128/1141 (11.2%)
Currently receiving outpatient treatment for mental health problems	31/289 (10.7%)	29/292 (9.9%)	35/296 (11.8%)	35/278 (12.6%)	130/1155 (11.3%)
Ever been hospitalized for mental health problems	17/286 (5.9%)	21/295 (7.1%)	13/295 (4.4%)	24/277 (8.7%)	75/1153 (6.5%)
Number of inpatient treatments for mental health problems	15.00 (5.15%)	18.00 (6.08%)	12.00 (4.00%)	22.00 (7.83%)	67.00 (5.74%)

*Note:* Description of the study population, age, gender, amount of kids with and without RD, mental health, and treatment; parents about themselves. Information from all parents is used.

Abbreviations: CF = CARE‐FAM, CF & WC = CARE‐FAM and WEP‐CARE, RD = rare disease, TAU = treatment as usual, WC = WEP‐CARE.

### 3.2 Analysis of Endpoints

Table 2 shows the results of the HRQoL analysis using the SF‐12. The treatment effects for the two SF‐12 scales vary between 0.10 (95% CI: [−0.69, 0.88]) for the mean difference between WEP‐CARE and TAU or CARE‐FAM, 0.66 (95% CI: [−0.13, 1.45]; expressed in measured score values) for the mean difference between CARE‐FAM and TAU or WEP‐CARE on the physical SF‐12 scale, and 0.14 (95% CI: [−0.93, 1.22]) for the mean difference between WEP‐CARE and TAU or CARE‐FAM and 0.66 (95% CI: [−0.42, 1.73]) for the mean difference between CARE‐FAM and TAU or WEP‐CARE on the SF‐12 mental scale, indicating a trend toward improvement in HRQoL in the intervention groups compared with the TAU group. Table 3 shows the results of the mental health analysis using the BSI, including all parents. The treatment effects range from trivial to small. For the total BSI score, the mean difference in the measured score between CARE‐FAM compared to TAU or WEP‐CARE alone was −0.20 (95% CI: [−1.81, 1.40]). When comparing WEP‐CARE with TAU and CARE‐FAM alone, different treatment effects were observed at different time points. After 6 months, the treatment effect was 0.99 (95% CI: [−0.86, 2.58]), after 12 months −1.66 (95% CI: [−3.60, 0.28]), and after 18 months −0.63 (95% CI: [−2.58, 1.31]). The intervention groups seem to work independently of each other. The time‐dependent shift in the parents' mental health from the treatment effects can be seen in the compulsivity mean of the BSI in the total population of all parents. Similar temporal changes can be seen in the mean aggressiveness and hostility scores on the BSI in the total population of all parents (Table 4). In addition, there are temporal effects on the BSI total scale endpoint and the BSI global score. A stable treatment effect on mental health and HRQoL over time as assessed by the BSI and the SF‐12 was not observed for all (sub)scales of the BSI (see Table 4). However, treatment effects according to the SF‐12 seemed to remain stable over time (at 12 months and at 18 months). Comparisons between all groups can be found in Tables S1 and S2.

**TABLE 2:** SF‐12—summary of total values of models with change from baseline, all (symptomatic and non‐symptomatic) parents.

Variable	Time	TAU			CF			WC			CF&WC			Comparison	Diff [95% CI]	Interaction *p*				
		*n*	*M* (SD)	adj. *M* [95% CI]	*n*	*M* (SD)	adj. *M* [95% CI]	*n*	*M* (SD)	adj. *M* [95% CI]	*n*	*M* (SD)	adj. *M* [95% CI]			*p*	CF‐time	WC‐time	CF‐WC‐time	CF‐WC
SF‐12 (physical)	T0	278	51.65 (7.92)		287	51.69 (8.98)		286	52.11 (8.42)	267	52.62 (7.64)						0.524	0.150	NA	0.260
	T2–T0	188	−0.25 (7.50)	−0.83 [−1.61, −0.05]	209	−0.12 (7.63)	−0.17 [−0.93, 0.59]	187	−0.43 (8.04)	−0.73 [−1.51, 0.04]	193	−0.74 (7.50)	−0.07 [−0.84, 0.70]							
	T3–T0	162	−1.50 (8.35)	−1.08 [−1.87, −0.29]	180	−0.18 (7.88)	−0.42 [−1.20, 0.36]	167	−0.41 (7.47)	−0.98 [−1.78, −0.19]	158	−0.57 (7.13)	−0.32 [−1.12, 0.48]							
	T4–T0	161	−1.96 (8.25)	−1.75 [−2.54, −0.96]	182	−0.27 (8.89)	−1.09 [−1.87, −0.31]	163	−1.65 (8.03)	−1.65 [−2.45, −0.86]	155	−1.74 (8.39)	−0.99 [−1.79, −0.19]							
	All													CF vs. other	0.66 [−0.13, 1.45]	0.099				
	All													WC vs. other	0.10 [−0.69, 0.88]	0.808				
SF‐12 (mental)	T0	278	44.69 (11.62)		287	43.33 (12.58)		286	43.83 (12.28)		267	43.61 (11.82)					0.602	0.733	NA	0.708
	T2–T0	188	0.67 (10.67)	1.59 [0.57, 2.62]	209	2.91 (11.56)	2.25 [1.24, 3.26]	187	1.17 (9.16)	1.74 [0.71, 2.77]	193	3.04 (9.36)	2.39 [1.36, 3.42]							
	T3–T0	162	2.47 (10.58)	2.78 [1.73, 3.83]	180	3.80 (12.05)	3.43 [2.39, 4.47]	167	3.06 (9.20)	2.92 [1.87, 3.98]	158	3.69 (10.22)	3.58 [2.51, 4.64]							
	T4–T0	161	3.01 (11.71)	3.19 [2.14, 4.25]	182	3.99 (11.60)	3.85 [2.81, 4.89]	163	3.48 (11.00)	3.34 [2.28, 4.40]	155	4.86 (11.06)	3.99 [2.93, 5.06]							
	All													CF vs. other	0.66 [−0.42, 1.73]	0.231				
	All													WC vs. other	0.14 [−0.93, 1.22]	0.792				

*Note:* Results of the health‐related quality of life of all parents and all groups over the four different time measurement points.

Abbreviations: adj. M = adjusted mean, CF = CARE‐FAM, CF & WC = CARE‐FAM and WEP‐CARE, CI = confidence interval, diff = difference, NA = not applicable, SF‐12 = Short Form 12; shortened version of the Short Form 36 questionnaire, TAU = treatment as usual, T0 = randomization, T1 = data 6 months after randomization, T2 = data 12 months after randomization, T3 = data 18 months after randomization, WC = WEP‐CARE.

**TABLE 3:** BSI—summary of total values of models with change from baseline, all (symptomatic and non‐symptomatic) parents.

Variable	Time	TAU			CF			WC			CF&WC			Com‐parison	Diff [95% CI]	Interaction *p*				
		*n*	*M* (SD)	adj. *M* [95% CI]	*n*	*M* (SD)	adj. *M* [95% CI]	*n*	*M* (SD)	adj. *M* [95% CI]	*n*	*M* (SD)	adj. *M* [95% CI]			*p*	CF‐time	WC‐time	CF‐WC‐time	CF‐WC
BSI—TS	T0	276	19.21 (19.23)		277	20.40 (19.84)		280	20.62 (22.56)		263	17.83 (16.99)					0.260	0.015	NA	0.467
	T2–T0	185	−1.92 (12.21)	−2.76 [−4.31, −1.21]	202	−4.36 (15.82)	−2.96 [−4.50, −1.43]	184	−1.74 (13.29)	−1.77 [−3.34, −0.20]	186	−1.75 (14.28)	−1.97 [−3.54, −0.41]	WC vs. other	0.99 [−0.86, 2.85]	0.294				
	T3–T0	167	−3.06 (13.28)	−3.12 [−4.72, −1.53]	172	−4.01 (14.75)	−3.33 [−4.91, −1.74]	168	−5.65 (12.78)	−4.79 [−6.40, −3.17]	151	−3.98 (12.92)	−4.99 [−6.62, −3.36]	WC vs. other	−1.66 [−3.60, 0.28]	0.093				
	T4–T0	166	−5.00 (14.63)	−4.37 [−5.96, −2.77]	176	−5.05 (13.65)	−4.57 [−6.15, −2.99]	160	−5.75 (14.98)	−5.00 [−6.62, −3.37]	150	−4.34 (15.95)	−5.20 [−6.84, −3.57]	WC vs. other	−0.63 [−2.58, 1.31]	0.524				
														CF vs. other	−0.20 [−1.81, 1.40]	0.803				
BSI—GSI	T0	276	0.36 (0.36)		277	0.38 (0.37)		280	0.39 (0.43)		263	0.34 (0.32)					0.260	0.015	NA	0.467
	T2–T0	185	−0.04 (0.23)	−0.05 [−0.08, −0.02]	202	−0.08 (0.30)	−0.06 [−0.08, −0.03]	184	−0.03 (0.25)	−0.03 [−0.06, −0.00]	186	−0.03 (0.27)	−0.04 [−0.07, −0.01]	WC vs. other	0.02 [−0.02, 0.05]	0.294				
	T3–T0	167	−0.06 (0.25)	−0.06 [−0.09, −0.03]	172	−0.08 (0.28)	−0.06 [−0.09, −0.03]	168	−0.11 (0.24)	−0.09 [−0.12, −0.06]	151	−0.08 (0.24)	−0.09 [−0.12, −0.06]	WC vs. other	−0.03 [−0.07, 0.01]	0.093				
	T4–T0	166	−0.09 (0.28)	−0.08 [−0.11, −0.05]	176	−0.10 (0.26)	−0.09 [−0.12, −0.06]	160	−0.11 (0.28)	−0.09 [−0.12, −0.06]	150	−0.08 (0.30)	−0.10 [−0.13, −0.07]	WC vs. other	−0.01 [−0.05, 0.02]	0.524				
	All													CF vs. other	−0.00 [−0.03, 0.03]	0.803				

*Note:* Results of the mental health measurement of all parents and all groups over the four different time measurement points.

Abbreviations: 95% CI = 95% confidence interval, adj. M = adjusted mean, BSI GSI = global characteristics value, BSI TS = Brief Symptom Inventory sum of the total values S1‐S10, CF = CARE‐FAM, CF & WC = CARE‐FAM and WEP‐CARE, TAU = treatment as usual, T0 = randomization, T1 = data 6 months after randomization, T2 = data 12 months after randomization, T3 = data 18 months after randomization, WC = WEP‐CARE.

**TABLE 4:** BSI—Models with change from baseline in subscales, all (symptomatic and non‐symptomatic) parents.

Variable	Time	TAU			CF			WC			CF & WC			Comparison	Diff [95% CI]	Interaction *p*				
		*n*	*M* (SD)	adj. *M* [95% CI]	*n*	*M* (SD)	adj. *M* [95% CI]	*n*	*M* (SD)	adj. *M* [95% CI]	*n*	*M* (SD)	adj. *M* [95% CI]			*p*	CF‐time	WC‐time	CF‐WC‐time	CF‐WC
BSI: Compulsiveness Mean	T0	285	0.59 (0.59)		293	0.61 (0.59)		295	0.63 (0.67)		278	0.59 (0.55)					0.483	0.021	NA	0.411
	T2–T0	193	−0.04 (0.46)	−0.09 [−0.14, −0.04]	216	−0.13 (0.49)	−0.09 [−0.14, −0.05]	197	−0.07 (0.43)	−0.06 [−0.11, −0.01]	206	−0.05 (0.44)	−0.06 [−0.11, −0.02]	WC vs. other	0.03 [−0.03, 0.09]	0.325				
	T3–T0	172	−0.08 (0.44)	−0.09 [−0.14, −0.03]	189	−0.10 (0.53)	−0.09 [−0.14, −0.04]	176	−0.17 (0.43)	−0.14 [−0.19, −0.09]	165	−0.11 (0.43)	−0.14 [−0.20, −0.09]	WC vs. other	−0.05 [−0.12, 0.01]	0.092				
	T4–T0	170	−0.13 (0.51)	−0.12 [−0.17, −0.07]	187	−0.14 (0.56)	−0.12 [−0.18, −0.07]	169	−0.16 (0.49)	−0.15 [−0.20, −0.10]	161	−0.15 (0.49)	−0.16 [−0.21, −0.10]	WC vs. other	−0.03 [−0.09, 0.03]	0.317				
	All													CF vs. other	−0.01 [−0.06, 0.04]	0.822				
BSI: Aggressiveness and hostility. Mean	T0	287	0.44 (0.48)		292	0.52 (0.56)		294	0.50 (0.55)		278	0.42 (0.42)					0.684	0.025	NA	0.130
	T2–T0	194	−0.04 (0.34)	−0.09 [−0.13, −0.05]	216	−0.17 (0.47)	−0.11 [−0.15, −0.07]	196	−0.09 (0.43)	−0.06 [−0.10, −0.02]	206	−0.04 (0.37)	−0.08 [−0.12, −0.04]	WC vs. other	0.03 [−0.02, 0.07]	0.247				
	T3–T0	173	−0.09 (0.37)	−0.10 [−0.14, −0.06]	189	−0.14 (0.50)	−0.12 [−0.16, −0.08]	176	−0.17 (0.40)	−0.14 [−0.18, −0.10]	166	−0.12 (0.41)	−0.16 [−0.20, −0.11]	WC vs. other	−0.04 [−0.09, 0.01]	0.127				
	T4–T0	170	−0.14 (0.41)	−0.15 [−0.19, −0.11]	190	−0.20 (0.47)	−0.17 [−0.21, −0.13]	167	−0.20 (0.44)	−0.18 [−0.22, −0.13]	160	−0.17 (0.40)	−0.19 [−0.24, −0.15]	WP vs. other	−0.03 [−0.08, 0.02]	0.271				
	All													CF vs. other	−0.02 [−0.06, 0.02]	0.354				

*Note:* Results of the mental health measurement of some subscales for all parents and all groups over the four different time measurement points.

Abbreviations: adj. *M* = adjusted mean, BSI = Brief Symptom Inventory, CF = CARE‐FAM, CF & WC = CARE‐FAM and WEP‐CARE, TAU = treatment as usual, T0 = randomization, T1 = data 6 months after randomization, T2 = data 12 months after randomization, T3 = data 18 months after randomization, WC = WEP‐CARE.

### 3.3 Additional Observations

Table 5 shows results for an analysis of mental health via the BSI, including only symptomatic parents (*T*‐value of the GSI of the BSI above 63 at baseline). Only small treatment effects between interventions and corresponding comparators can be seen, and the corresponding CIs include zero. The treatment effect varied between −3.74 (95% CI: [−13.14, 5.65]) and 8.58 (95% CI: [−0.50, 17.66]), showing non‐relevant differences between conditions.

**TABLE 5:** BSI—summary of total values of models with change from baseline, only symptomatic parents about themselves.

Variable	Time	TAU			CF			WC			CF&WC			Com‐parison	Diff [95% CI]	Interaction *p*				
		*n*	*M* (SD)	adj. *M* [95% CI]	*n*	*M* (SD)	adj. *M* [95% CI]	*n*	*M* (SD)	adj. *M* [95% CI]	*n*	*M* (SD)	adj. *M* [95% CI]			*p*	CF‐time	WC‐time	CF‐WC‐time	CF‐WC
BSI—TS	T0	67	44.37 (21.22)		76	46.12 (17.98)		57	53.93 (28.11)		52	43.54 (17.49)					0.455	0.020	NA	0.814
	T2–T0	27	−14.67 (17.14)	−13.71 [−20.9, −6.56]	46	−12.91 (26.84)	−13.99 [−20.5, −7.51]	23	−8.13 (29.92)	−5.12 [−13.07, 2.82]	17	−11.47 (24.37)	−5.41 [−13.8, 2.97]	WC vs. other	8.58 [−0.50, 17.7]	0.064				
	T3−T0	26	−12.46 (22.76)	−11.42 [−18.6, −4.20]	40	−9.38 (24.45)	−11.70 [−18.4, −5.06]	21	−17.48 (26.73)	−15.16 [−23.4, −6.96]	14	−19.07 (24.53)	−15.45 [−24.1, −6.76]	WC vs. other	−3.74 [−13.1, 5.65]	0.433				
	T4–T0	30	−20.43 (18.98)	−15.41 [−22.6, −8.26]	39	−12.41 (21.32)	−15.70 [−22.4, −9.05]	23	−17.65 (32.69)	−16.53 [−24.5, −8.58]	17	−20.71 (32.78)	−16.82 [−25.2, −8.40]	WC vs. other	−1.12 [−10.3, 8.02]	0.810				
	All													CF vs. other	−0.29 [−7.98, 7.40]	0.941				
BSI—GSI	T0	67	0.84 (0.40)		76	0.87 (0.34)		57	1.02 (0.53)		52	0.82 (0.33)					0.455	0.020	NA	0.814
	T2–T0	27	−0.28 (0.32)	−0.26 [−0.39, −0.12]	46	−0.24 (0.51)	−0.26 [−0.39, −0.14]	23	−0.15 (0.56)	−0.10 [−0.25, 0.05]	17	−0.22 (0.46)	−0.10 [−0.26, 0.06]	WC vs. other	0.16 [−0.01, 0.33]	0.064				
	T3–T0	26	−0.24 (0.43)	−0.22 [−0.35, −0.08]	40	−0.18 (0.46)	−0.22 [−0.35, −0.10]	21	−0.33 (0.50)	−0.29 [−0.44, −0.13]	14	−0.36 (0.46)	−0.29 [−0.46, −0.13]	WC vs. other	−0.07 [−0.25, 0.11]	0.433				
	T4–T0	30	−0.39 (0.36)	−0.29 [−0.43, −0.16]	39	−0.23 (0.40)	−0.30 [−0.42, −0.17]	23	−0.33 (0.62)	−0.31 [−0.46, −0.16]	17	−0.39 (0.62)	−0.32 [−0.48, −0.16]	WC vs. other	−0.02 [−0.19, 0.15]	0.810				
	All													CF vs. other	−0.01 [−0.15, 0.14]	0.941				

*Note:* Results of the mental health measurement of the symptomatic parents and all groups over the four different time measurement points.

Abbreviations: adj. M = adjusted mean, BSI GSI = Global characteristics value, BSI TS = Brief Symptom Inventory sum of the total values S1‐S10, CF = CARE‐FAM, CF & WC = CARE‐FAM and WEP‐CARE, TAU = treatment as usual, T0 = randomization, T1 = data 6 months after randomization, T2 = data 12 months after randomization, T3 = data 18 months after randomization, WC = WEP‐CARE.

Beyond the established hypotheses, the PHQ‐9 and ULQIE are of interest for the assessment of mental health and HRQoL to ensure that the results obtained from the BSI and the SF‐12 are constant across different instruments. Table 6 shows the results of the analysis of depression as measured by the PHQ‐9 and HRQoL as measured by the ULQIE. The results for parental depression symptoms via the PHQ‐9 show small treatment effects for the comparison within factor variables for the treatment groups. The effects vary between small and medium treatment effects. Detailed information on these results can be found in Table 5. For the PHQ‐9, the treatment effect for CARE‐FAM compared to TAU or WEP‐CARE alone is −0.14 (95% CI: [−0.50, 0.21]), and for WEP‐CARE compared to TAU or CARE‐FAM alone is −0.03 (95% CI: [−0.38, 0.33]), showing that the improvement in the interventions is greater than in TAU. Specifically, for depression symptoms, the effect for CARE‐FAM compared to TAU and WEP‐CARE alone shows a small treatment effect, as well as for WEP‐CARE compared to TAU and CARE‐FAM. The results again show that the improvement is greater with the interventions than with TAU. For some scales of HRQoL via the ULQIE, the assumption of independent interventions does not seem to be fulfilled. The total score shows an effect of 0.01 (95% CI: [−0.05, 0.06]) for CARE‐FAM compared to TAU and no treatment effect in the WEP‐CARE group. Furthermore, the treatment effect does not seem to be stable over time. Comparisons between all groups can be found in the Supporting Information.

**TABLE 6:** PHQ‐9 and ULQIE—summary of total values of models with change from baseline, all (symptomatic and non‐symptomatic) parents.

Variable	Time	TAU			*n*	CF	adj. *M* [95% CI]	WC			CF & WC			Comparison	Diff [95% CI]	*p*	Interaction *p*			
		*n*	*M* (SD)	adj. *M* [95% CI]		*M* (SD)		*n*	*M* (SD)	adj. *M* [95% CI]	*n*	*M* (SD)	adj. *M* [95% CI]				CF‐time	WC‐time	CF‐WC‐time	CF‐WC
PHQ‐9 total value	T0	289	5.89 (4.52)		296	6.07 (4.63)		298	6.15 (4.57)		280	5.74 (4.30)					0.859	0.213	NA	0.267
	T2–T0	195	−0.67 (3.70)	−0.74 [−1.08, −0.40]	222	−1.05 (3.56)	−0.88 [−1.21, −0.55]	197	−0.73 (3.01)	−0.77 [−1.10, −0.43]	210	−0.76 (3.31)	−0.91 [−1.24, −0.58]							
	T3–T0	175	−0.62 (3.57)	−0.98 [−1.32, −0.64]	192	−1.41 (3.44)	−1.12 [−1.46, −0.79]	177	−1.31 (3.22)	−1.01 [−1.35, −0.67]	168	−0.83 (3.21)	−1.15 [−1.50, −0.81]							
	T4–T0	173	−1.17 (4.10)	−1.27 [−1.61, −0.93]	190	−1.38 (3.75)	−1.41 [−1.75, −1.07]	171	−1.52 (3.48)	−1.30 [−1.64, −0.95]	161	−1.40 (3.60)	−1.44 [−1.79, −1.09]							
	All													CF vs. other	−0.14 [−0.50, 0.21]	0.434				
	All													WC vs. other	−0.03 [−0.38, 0.33]	0.881				
ULQIE: total score	T0	263	2.58 (0.61)		266	2.55 (0.61)		266	2.57 (0.55)		252	2.56 (0.55)					0.742	0.517	NA	0.072
	T2–T0	170	0.03 (0.40)	0.06 [0.00, 0.11]	177	0.12 (0.44)	0.06 [0.01, 0.12]	168	0.08 (0.45)	0.06 [0.00, 0.11]	175	0.03 (0.46)	0.06 [0.01, 0.12]							
	T3–T0	151	0.08 (0.43)	0.12 [0.06, 0.17]	158	0.15 (0.44)	0.12 [0.07, 0.18]	152	0.15 (0.41)	0.12 [0.06, 0.17]	143	0.11 (0.43)	0.12 [0.07, 0.18]							
	T4–T0	153	0.12 (0.48)	0.14 [0.08, 0.19]	151	0.16 (0.42)	0.14 [0.09, 0.20]	146	0.18 (0.46)	0.14 [0.08, 0.19]	142	0.13 (0.46)	0.14 [0.09, 0.20]							
	All													CF vs. other	0.01 [−0.05, 0.06]	0.821				
	All													WC vs. other	0.00 [−0.05, 0.05]	0.993				

*Note:* Results of the health‐related quality of life and the mental health measurement of all parents and all groups over the four different time measurement points.

Abbreviations: adj. *M* = adjusted mean, CF = CARE‐FAM, CF & WC = CARE‐FAM and WEP‐CARE, PHQ‐9 = Patient Health Questionnaire‐9, TAU = treatment as usual, T0 = randomization, T1 = data 6 months after randomization, T2 = data 12 months after randomization, T3 = data 18 months after randomization, ULQIE = Ulm Quality of Life Inventory for Parents of Chronically Ill Children, WC = WEP‐CARE.

## 4 Discussion

This study evaluated the effectiveness of two family‐based interventions for families of children with RDs on parental HRQoL and mental health. The randomized controlled design allowed the creation of structurally similar groups with respect to known and unknown potential confounders. A comparison was made with TAU, i.e., clinical routine.

No clinically significant differences were observed between the treatment groups in the HrQoL (SF‐12) of the affected parents, and the effects remained stable over time. Similarly, no clinically significant differences were observed between treatment groups in parents' mental health (BSI), and these effects also remained largely stable over time. Analysis of HRQoL using the SF‐12 showed a trend toward improvement in the intervention groups compared with the TAU group, both on the physical and mental scales. Although such a result was initially hypothesized, only tendencies could be found, so the hypothesis cannot be confirmed. Nevertheless, a slight improvement in parental HRQoL over time is promising. The analysis of mental health using the BSI showed that treatment effects ranged from trivial to small. Differences were observed at different time points, although the intervention groups appeared to operate independently. Again, treatment effects were found that demonstrated the use of the interventions developed. Although the results showed no relevant differences between conditions, the hypothesis regarding parents' mental health was supported by the results. Time‐dependent changes in parents' mental health status were observed on several BSI scales. A stable treatment effect over time could not be observed for all scales of the BSI, whereas treatment effects on the SF‐12 remained stable over time. Furthermore, the assumption of independence of the interventions does not seem to be fulfilled for a small number of secondary outcomes, and the assumption of constant intervention effects does not seem to be fulfilled for all outcomes. The additional analysis of other outcomes is also inconclusive. The hypotheses that all parents (both symptomatic and non‐symptomatic) in the intervention groups would have higher HRQoL, fewer psychological symptoms, and improved mental health compared to TAU at 6 months, and that these differences would remain stable at 12 and 18 months, cannot be confirmed.

However, it is clear that affected parents are particularly burdened in terms of their HRQoL and mental health, which is consistent with the findings of previous research in these areas (Boettcher et al. 2021; Rihm et al. 2022). The interventions evaluated can still help burdened parents improve their HRQoL and mental health and are therefore crucial in supporting affected families, which is urgently needed and has been called for in previous research (Frank et al. 2014; Boettcher et al. 2021; Fuerboeter et al. 2021).

One reason for the not relevant differences in the results may be the COVID‐19 pandemic, which coincided with the time when the study was carried out and may have exacerbated the challenges faced by parents of children with RDs, potentially impacting their HRQoL and mental health. Families of children with (Fuerboeter et al. 2021; Boettcher et al. 2022) and without RDs (Ravens‐Sieberer et al. 2021; Gurdasani et al. 2021) were significantly affected by the disruption to daily life caused by the COVID‐19 pandemic. Parents of children with RD who already feel socially isolated may have experienced increased isolation during the pandemic, potentially worsening their well‐being (Witt et al. 2023; Chung et al. 2020). To understand the results, it is important to note that over 30% of families receiving the CARE‐FAM intervention eventually received the intervention in an online setting or by telephone, a change that had to be implemented due to the COVID‐19 pandemic. The intervention was developed as a face‐to‐face treatment and was not evaluated in advance for the telephone or web setting. These unplanned changes may have influenced the results. An additional factor in the results may have been that there was a delay in the start of the WEP‐CARE intervention, which meant that not all families were able to complete the intervention within the first 6 months. Failure to complete the treatment may have led to unexpected results. The ambiguous results may also be due to patients being more aware of their problem or focusing more on their symptoms at the start of treatment. This may lead to an apparent worsening of symptoms that is not necessarily an actual worsening but rather the result of increased awareness. As a result, patients may report their symptoms as more severe at the start of treatment, which may have biased the baseline results. Eight sessions in a family setting may therefore be too few for the individual to be able to absorb their difficulties and deal with them therapeutically. In addition, it should be noted that the selection of families was too liberal and the burden of illness in the families was not high enough to achieve significant effects, as illness severity was not an inclusion criterion and was not categorized.

On the basis of the available results on the influence of the CARE‐FAM, WEP‐CARE, and CARE‐FAM + WEP‐CARE interventions on HRQoL and parental mental health alone, no clear decision can yet be made about the inclusion of the intervention in the standard care of the family therapies evaluated in CARE‐FAM‐NET. Also important for this decision is the effect on the health of the children with RD, affected siblings (also part of the family constellation) and the cost–benefit effect; i.e., the health economic analysis should be considered here. Further analysis is needed before final decisions can be made.

In addition to the results, it is important to mention that such a study has never been conducted in Germany before and therefore represents a major step forward for family‐based interventions as well as for research. To the best of our knowledge, only one pilot study has so far investigated a family intervention, with promising results (Lyon et al. 2024). However, the CARE‐FAM‐NET study examined a much larger population, which may provide new insights for working with affected families and providing them with the necessary psychosocial support.

### 4.1 Strengths and Limitations

There are many positive aspects. The study was pre‐registered in international registries before data collection began and a study protocol was published. A statistical analysis plan for the evaluation of effects was prepared by the before the data were available. An elaborate RCT was conducted with a sufficiently large number of enrolled families to ensure that structurally similar groups were created with regard to known and, above all, unknown potential confounding variables. This ensures that no factors interfere with the comparison of the new care approaches. Despite the challenging conditions created by the COVID‐19 pandemic, a sufficiently large sample of families was successfully enrolled in the study. The present evaluation of the interventions was conducted under very strict methodological conditions, including a high level of adherence to the study protocol. This is evidenced by several features: (a) effective blinding of interviewers at all stages of the study, (b) all interviews and external evaluations were conducted by a qualified team, (c) the evaluating biometric institute conducted a blinded statistical analysis of group membership.

The following deviations from the study protocol occurred. Due to the COVID‐19 pandemic, some limitations were observed, which were addressed in further sensitivity analyses. The minimum number of completed sessions of the WEP‐CARE intervention was reduced from 12 to 6 writing tasks in order to be able to say that the intervention had been successfully completed. The regular number of completed writing tasks was 12 to ensure that the intervention was delivered according to protocol. Instead of using the recruitment center as a fixed effect, it was included as a random effect in all models to simplify parameter estimation. Potential limitations that need to be discussed include the following: For some secondary endpoints, a lack of observations did not allow us to draw valid conclusions. In addition, the control group received a clinical interview at baseline, which could be seen as a follow‐up intervention or may have led the participating parents to seek socio‐psychological support outside of this study. This could explain why the control groups had better baseline scores than expected and why the differences at 6 and 12 months were not as significant as expected.

## 5 Conclusion

First, it should be noted that the evaluation of two innovative care approaches (CARE‐FAM and WEP‐CARE) for the treatment of children with RDs and their families in routine care, with the aim of being more cost‐effective and effective, as well as having a positive impact on HRQoL and mental health, was successfully carried out. Due to unforeseen events, minor changes had to be made to the program. A comparison was made with TAU as a control condition, which significantly reduced the likelihood of detecting major differences in the endpoints analyzed. Nevertheless, if the results are consistent with the hypothesis, it will be possible to demonstrate high relevance to care. The study did not investigate a specific indication group, but included families with children with a wide range of RDs that are highly relevant to care. Despite the challenges posed by the COVID‐19 pandemic, it was possible to recruit a sufficiently large sample of families for the study. The evaluation of the care models was carried out in strict compliance with the study protocol, randomization, and other methodological standards. The results show trends in treatment effects that suggest improvements in quality of life and mental health and indicate that the assumptions underlying the present analyses are reasonable and should be investigated further. Several relevant aspects must be considered as potential limitations of the present study. These include minor but necessary changes to the study protocol. In addition, for some outcomes, too few events were observed to draw valid conclusions.

## Conflicts of Interest

The authors declare no conflicts of interest.

## Supporting information


**Table S1.** CARE‐FAM‐NET: all comparisons, all parents.
**Table S2.** CARE‐FAM‐NET: only distressed parents.

## Data Availability

The dataset was stored by the Hamburg study center, which coordinated the overall study. The Institute of Medical Biometry of the Hamburg study center analyzed the data. The independent research organization CTC North, based in Hamburg, Germany, managed the data and the monitoring. Data handling followed the EU General Data Protection Regulation (DSGVO), and the data will be stored for at least 10 years. On reasonable request, the data can be provided by the last author.

## References

[famp70041-bib-0002] Baumbusch, J. , S. Mayer , and I. Sloan‐Yip . 2018. “Alone in a Crowd? Parents of Children With Rare Diseases' Experiences of Navigating the Healthcare System.” Journal of Genetic Counseling 28: 80–90. 10.1007/s10897-018-0294-9. 30128673

[famp70041-bib-0004] Boettcher, J. , M. Boettcher , S. Wiegand‐Grefe , and H. Zapf . 2021. “Being the Pillar for Children With Rare Diseases—A Systematic Review on Parental Quality of Life.” International Journal of Environmental Research and Public Health 18, no. 9: 4993. 10.3390/ijerph18094993. 34066738 PMC8125857

[famp70041-bib-0005] Boettcher, J. , B. Filter , J. Denecke , et al. 2020. “Evaluation of Two Family‐Based Intervention Programs for Children Affected by Rare Disease and Their Families—Research Network (CARE‐FAM‐NET): Study Protocol for a Rater‐Blinded, Randomized, Controlled, Multicenter Trial in a 2 × 2 Factorial Design.” BMC Family Practice 21, no. 1: 239. 10.1186/s12875-020-01312-9. 33218310 PMC7678588

[famp70041-bib-0006] Boettcher, J. , R. Nazarian , M. Fuerboeter , et al. 2022. “Mental Health of Siblings of Children With Rare Congential Surgical Diseases During the COVID‐19 Pandemic.” European Journal of Pediatric Surgery 32, no. 5: 422–428. 10.1055/s-0041-1740978. 34972234

[famp70041-bib-0007] Candy, B. , L. Jones , R. Drake , B. Leurent , and M. King . 2011. “Interventions for Supporting Informal Caregivers of Patients in the Terminal Phase of a Disease.” Cochrane Database of Systematic Reviews 6: Cd007617. 10.1002/14651858.CD007617.pub2. PMC13247825 21678368

[famp70041-bib-0008] Chung, C. C. , W. H. Wong , J. L. Fung , R. D. Hong Kong , and B. H. Chung . 2020. “Impact of COVID‐19 Pandemic on Patients With Rare Disease in Hong Kong.” European Journal of Medical Genetics 2020, no. 63: 337–339. 10.1016/j.ejmg.2020.104062 PMC7486880 32931946

[famp70041-bib-0009] Cohn, L. N. , P. Pechlivanoglou , Y. Lee , et al. 2020. “Health Outcomes of Parents of Children With Chronic Illness: A Systematic Review and Meta‐Analysis.” Journal of Pediatrics 218: 166–177. 10.1016/j.jpeds.2019.10.068. 31916997

[famp70041-bib-0011] Derogatis, L. R. , and H. Franke . 2000. Brief Symptom Inventory (Kurzform der SCL‐90‐R). Deutsche Version. Beltz Test Gesellschaft.

[famp70041-bib-0012] Dong, D. , R. Y.‐N. Chung , R. H. W. Chan , S. Gong , and R. H. Xu . 2020. “Why Is Misdiagnosis More Likely Among Some People With Rare Diseases Than Others? Insights From a Population‐Based Cross‐Sectional Study in China.” Orphanet Journal of Rare Diseases 15, no. 1: 307. 10.1186/s13023-020-01587-2. 33115515 PMC7594300

[famp70041-bib-0013] Fidika, A. , M. Herle , C. Lehmann , C. Weiss , C. Knaevelsrud , and L. Goldbeck . 2015. “A Web‐Based Psychological Support Program for Caregivers of Children With Cystic Fibrosis: A Pilot Study.” Health and Quality of Life Outcomes 13, no. 1: 11. 10.1186/s12955-015-0211-y. 25652684 PMC4336741

[famp70041-bib-0014] Frank, M. , D. Eidt‐Koch , I. Aumann , A. Reimann , T. O. F. Wagner , and J. M. von der Schulenburg . 2014. “Maßnahmen zur Verbesserung der Gesundheitlichen Situation von Menschen mit Seltenen Erkrankungen in Deutschland.” Bundesgesundheitsblatt, Gesundheitsforschung, Gesundheitsschutz 57, no. 10: 1216–1223. http://europa.eu.int/comm/health/ph_threats/non_com/rare_diseases_en.htm. 25209683 10.1007/s00103-014-2040-2

[famp70041-bib-0015] Fuerboeter, M. , J. Boettcher , C. Barkmann , et al. 2021. “Quality of Life and Mental Health of Children With Rare Congenital Surgical Diseases and Their Parents During the COVID‐19 Pandemic.” Orphanet Journal of Rare Diseases 16: 498. 10.1186/s13023-021-02129-0. 34838064 PMC8626760

[famp70041-bib-0016] Goldbeck, L. , and M. Storck . 2002. “Das Ulmer Lebensqualitäts‐Inventar für Eltern Chronisch Kranker Kinder (ULQIE).” Zeitschrift für Klinische Psychologie und Psychotherapie 31, no. 1: 31–39.

[famp70041-bib-0038] Gräfe, K. , S. Zipfel , W. Herzog , and B. Löwe . 2004. “Screening psychischer Störungen mit dem“Gesundheitsfragebogen für Patienten (PHQ‐D)”.” Diagnostica 50: 171–181. 10.1026/0012-1924.50.4.171.

[famp70041-bib-0017] Gurdasani, D. , N. A. Alwan , T. Greenhalgh , et al. 2021. “School Reopening Without Robust COVID‐19 Mitigation Risks Accelerating the Pandemic.” Lancet 397: 1177–1178. 33713595 10.1016/S0140-6736(21)00622-X PMC9755467

[famp70041-bib-0018] Kirby, T. 2012. “Australia Makes Up for Lost Time on Rare Diseases.” Lancet 379, no. 9827: 1689–1690. 10.1016/s0140-6736(12)60702-8. 22567671

[famp70041-bib-0039] Kroenke, K. , R. L. Spitzer , and J. B. Williams . 2001. “The PHQ‐9: Validity of a Brief Depression Severity Measure.” Journal of General Internal Medicine 16, no. 9: 606–613. 11556941 10.1046/j.1525-1497.2001.016009606.x PMC1495268

[famp70041-bib-0019] Löwe, B. , R. L. Spitzer , S. Zipfel , and W. Herzog . 2002. Gesundheitsfragebogen für Patienten (PHQ‐D). Komplettversion und Kurzform. Autorisierte Deutsche Version des “Prime MD Patient Health Questionnaire (PHQ)”. Pfizer.

[famp70041-bib-0020] Lyon, M. E. , J. L. Fraser , J. D. Thompkins , et al. 2024. “Advance Care Planning for Children With Rare Diseases: A Pilot RCT.” Pediatrics 153, no. 6: e2023064557. 10.1542/peds.2023-064557. 38699801 PMC11153326

[famp70041-bib-0021] Mattejat, F. , C. Wüthrich , and H. Remschmidt . 2000. “Children of Parents of Psychiatric Disorders. Examplary Discusses of Research Perspectives in Invastigations of Children With Depressiv Parents.” Der Nervenarzt 71: 164–172. 10.1007/s001150050025. 10756524

[famp70041-bib-0022] McConkie‐Rosell, A. , S. R. Hooper , L. D. M. Pena , et al. 2018. “Psychosocial Profiles of Parents of Children With Undiagnosed Diseases: Managing Well or Just Managing?” Journal of Genetic Counseling 27, no. 4: 935–946. 10.1007/s10897-017-0193-5. 29297108 PMC6028305

[famp70041-bib-0023] McMullan, J. , A. L. Crowe , C. Bailie , et al. 2020. “Improvements Needed to Support People Living and Working With a Rare Disease in Northern Ireland: Current Rare Disease Support Perceived as Inadequate.” Orphanet Journal of Rare Diseases 15, no. 1: 315. 10.1186/s13023-020-01559-6. 33168042 PMC7649905

[famp70041-bib-0024] Moliner, A. M. , and J. Waligora . 2017. “The European Union Policy in the Field of Rare Diseases.” Advances in Experimental Medicine and Biology 1031: 561–587. 29214592 10.1007/978-3-319-67144-4_30

[famp70041-bib-0025] Morfeld, M. , I. Kirchberger , and M. Bullinger . 2011. “2 Ergänzte und Überarbeitete Auflage.” In SF‐36, Fragebogen zum Gesundheitszustand. Hogrefe.

[famp70041-bib-0026] Mundlos, C. 2017. “Bessere Versorgungsstrukturen für Seltene Erkrankungen.” Monatsschrift für Kinderheilkunde 165: 202–210. 10.1007/s00112-016-0224-6.

[famp70041-bib-0027] Pelentsov, L. J. , A. L. Fielder , T. A. Laws , and A. J. Esterman . 2016. “The Supportive Care Needs of Parents With a Child With a Rare Disease: Results of an Online Survey.” BMC Family Practice 17, no. 1: 88. 27439905 10.1186/s12875-016-0488-x PMC4955113

[famp70041-bib-0028] Ravens‐Sieberer, U. , A. Kaman , M. Erhart , J. Devine , R. Schlack , and C. Otto . 2021. “Impact of the COVID‐19 Pandemic on Quality of Life and Mental Health in Children and Adolescents in Germany.” European Child & Adolescent Psychiatry 2021, no. 1: 3. 10.1007/s00787-021-01726-5 PMC7829493 33492480

[famp70041-bib-0029] Rihm, L. , M. Dreier , F. Rezvani , S. Wiegand‐Grefe , and J. Dirmaier . 2022. “The Psychosocial Situation of Families Caring for Children With Rare Diseases During the COVID‐19 Pandemic: Results of a Cross‐Sectional Online Survey.” Orphanet Journal of Rare Diseases 17, no. 1: 449. 36572906 10.1186/s13023-022-02595-0 PMC9791975

[famp70041-bib-0040] Senn, S. 1994. “Testing for baseline balance in clinical trials.” Statistics in medicine 13, no. 17: 1715–1726. 7997705 10.1002/sim.4780131703

[famp70041-bib-0031] Vandeborne, L. , E. van Overbeeke , M. Dooms , B. De Beleyr , and I. Huys . 2019. “Information Needs of Physicians Regarding the Diagnosis of Rare Diseases: A Questionnaire‐Based Study in Belgium.” Orphanet Journal of Rare Diseases 14, no. 1: 99. 10.1186/s13023-019-1075-8. 31054581 PMC6500578

[famp70041-bib-0032] Ware, J. E. , M. Kosinski , and S. D. Keller . 1996. “A 12‐Item Short‐Form Health Survey:Construction of Scales and Preliminary Tests of Reliability and Validity.” Medical Care 34, no. 3: 220–233. 8628042 10.1097/00005650-199603000-00003

[famp70041-bib-0033] Wiegand‐Grefe, S. , S. Halverscheid , and A. Plass . 2011. Kinder und Ihre Psychisch Kranken Eltern: Familienorientierte Prävention‐der CHIMPs‐Beratungsansatz. Hogrefe.

[famp70041-bib-0035] Witt, S. , K. Schuett , S. Wiegand‐Grefe , J. Boettcher , and J. Quitmann . 2023. “Living With a Rare Disease—Experiences and Needs in Pediatric Patients and Their Parents.” Orphanet Journal of Rare Diseases 18, no. 1: 242. 10.1186/s13023-023-02837-9. 37568186 PMC10422846

[famp70041-bib-0037] Zurynski, Y. , K. Frith , H. Leonard , and E. Elliott . 2008. “Rare Childhood Diseases: How Should We Respond?” Archives of Disease in Childhood 93, no. 12: 1071–1074. 10.1136/adc.2007.134940. 18684747

