# Supplementary material for: Evaluation of the Health‐Related Quality of Life and Mental Health of Parents With Children and Adolescents With a Rare Disease Based on the Results of a Randomized Controlled Trial to Investigate a Family‐Based Intervention and an Online Intervention for Affected Families (CARE‐FAM‐NET)
Source: Fam Process. 2025 May 15;64(2):e70041. doi: 10.1111/famp.70041 (PMC12081942; doi:10.1111/famp.70041)
Supplement: Supplementary file 1 — Table S1. CARE‐FAM‐NET: all comparisons, all parents. Table S2. CARE‐FAM‐NET: only distressed parents. [file FAMP-64-0-s001.docx]

**Supplemental Materials**

**Table S1**

*CARE-FAM-NET: All Comparisons, All Parents*

|  | | **TAU** | | | **CF** | | | **WC** | | | **CF&WC** | | |  | | | **Interaction p-value** | | | |
| --- | --- | --- | --- | --- | --- | --- | --- | --- | --- | --- | --- | --- | --- | --- | --- | --- | --- | --- | --- | --- |
| **Variable** | **Time** | ***n*** | ***M* (*SD*)** | **adj. *M* [95%CI]** | ***n*** | ***M* (*SD*)** | **adj. *M* [95%CI]** | ***n*** | ***M* (*SD*)** | **adj. *M* [95%CI]** | ***n*** | ***M* (*SD*)** | **adj. *M* [95%CI]** | **Compa-**  **rison** | **Diff [95%CI]** | ***p*** | **CF-time** | **WC-time** | **CF-WC-time** | **CF-WC** |
| SF-12 (Physical) | T0 | 278 | 51.65 (7.92) |  | 287 | 51.69 (8.98) |  | 286 | 52.11 (8.42) |  | 267 | 52.62 (7.64) |  |  |  |  | 0.52 | 0.15 | NA | 0.260 |
|  | T2-T0 | 188 | -0.25 (7.50) | -0.83  [-1.61-0.05] | 209 | -0.12 (7.63) | -0.17  [-0.93,0.59] | 187 | -0.43 (8.04) | -0.73  [-1.51, 0.04] | 193 | -0.74 (7.50) | -0.07  [-0.84, 0.70] |  |  |  |  |  |  |  |
|  | T3-T0 | 162 | -1.50 (8.35) | -1.08  [-1.87-0.29] | 180 | -0.18 (7.88) | -0.42  [-1.20,0.36] | 167 | -0.41 (7.47) | -0.98  [-1.78, -0.19] | 158 | -0.57 (7.13) | -0.32  [-1.12, 0.48] |  |  |  |  |  |  |  |
|  | T4-T0 | 161 | -1.96 (8.25) | -1.75  [-2.54-0.96] | 182 | -0.27 (8.89) | -1.09  [-1.87,-0.31] | 163 | -1.65 (8.03) | -1.65  [-2.45, -0.86] | 155 | -1.74 (8.39) | -0.99  [-1.79, -0.19] |  |  |  |  |  |  |  |
|  | All |  |  |  |  |  |  |  |  |  |  |  |  | CF vs TAU | 0.66  [-0.13, 1.45] | .10 |  |  |  |  |
|  | All |  |  |  |  |  |  |  |  |  |  |  |  | WC vs TAU | 0.10  [-0.69, 0.88] | .81 |  |  |  |  |
|  | All |  |  |  |  |  |  |  |  |  |  |  |  | WC vs CF | -0.56  [-1.67, 0.54] | .31 |  |  |  |  |
|  | All |  |  |  |  |  |  |  |  |  |  |  |  | WC & CF vs TAU | 0.76  [-0.37, 1.88] | .19 |  |  |  |  |
|  | All |  |  |  |  |  |  |  |  |  |  |  |  | WC & CF vs CF | 0.10  [-0.69, 0.88] | .81 |  |  |  |  |
|  | All |  |  |  |  |  |  |  |  |  |  |  |  | WC & CF vs WC | 0.66  [-0.13, 1.45] | .10 |  |  |  |  |
| SF-12 (Mental) | T0 | 278 | 44.69 (11.62) |  | 287 | 43.33 (12.58) |  | 286 | 43.83 (12.28) |  | 267 | 43.61 (11.82) |  |  |  |  | 0.60 | 0.73 | NA | 0.708 |
|  | T2-T0 | 188 | 0.67 (10.67) | 1.59  [0.57, 2.62] | 209 | 2.91 (11.56) | 2.25  [1.24, 3.26] | 187 | 1.17 (9.16) | 1.74  [0.71, 2.77] | 193 | 3.04 (9.36) | 2.39  [1.36, 3.42] |  |  |  |  |  |  |  |
|  | T3-T0 | 162 | 2.47 (10.58) | 2.78  [1.73, 3.83] | 180 | 3.80 (12.05) | 3.43  [2.39, 4.47] | 167 | 3.06 (9.20) | 2.92  [1.87, 3.98] | 158 | 3.69 (10.22) | 3.58  [2.51, 4.64] |  |  |  |  |  |  |  |
|  | T4-T0 | 161 | 3.01 (11.71) | 3.19  [2.14, 4.25] | 182 | 3.99 (11.60) | 3.85  [2.81, 4.89] | 163 | 3.48 (11.00) | 3.34  [2.28, 4.40] | 155 | 4.86 (11.06) | 3.99  2.93, 5.06] |  |  |  |  |  |  |  |
|  | All |  |  |  |  |  |  |  |  |  |  |  |  | CF vs TAU | 0.66  [-0.42, 1.73] | .23 |  |  |  |  |
|  | All |  |  |  |  |  |  |  |  |  |  |  |  | WC vs TAU | 0.14  [-0.93, 1.22] | .79 |  |  |  |  |
|  | All |  |  |  |  |  |  |  |  |  |  |  |  | WC vs CF | -0.51  [-2.01, 0.99] | .50 |  |  |  |  |
|  | All |  |  |  |  |  |  |  |  |  |  |  |  | WC & CF vs TAU | 0.80  [-0.73, 2.33] | .31 |  |  |  |  |
|  | All |  |  |  |  |  |  |  |  |  |  |  |  | WC & CF vs CF | 0.14  [-0.93, 1.22] | .79 |  |  |  |  |
|  | All |  |  |  |  |  |  |  |  |  |  |  |  | WC & CF vs WC | 0.66  [-0.42, 1.73] | .23 |  |  |  |  |
| BSI - TS | T0 | 276 | 19.21 (19.23) |  | 277 | 20.40 (19.84) |  | 280 | 20.62 (22.56) |  | 263 | 17.83 (16.99) |  |  |  |  | 0.26 | 0.01 | NA | 0.467 |
|  | T2-T0 | 185 | -1.92 (12.21) | -2.76  [-4.31,1.21] | 202 | -4.36 (15.82) | -2.96  [-4.5,-1.43] | 184 | -1.74 (13.29) | -1.77  [-3.34, -0.20] | 186 | -1.75 (14.28) | -1.97  [-3.54,-0.41] | CF vs TAU | -0.20  [-1.81, 1.40] | .80 |  |  |  |  |
|  | T2-T0 |  |  |  |  |  |  |  |  |  |  |  |  | WC vs TAU | 0.99  [-0.86, 2.85] | .29 |  |  |  |  |
|  | T2-T0 |  |  |  |  |  |  |  |  |  |  |  |  | WC vs CF | 1.20  [-1.24, 3.64] | .34 |  |  |  |  |
|  | T2-T0 |  |  |  |  |  |  |  |  |  |  |  |  | WC & CF vs TAU | 0.79  [-1.67, 3.25] | .53 |  |  |  |  |
|  | T2-T0 |  |  |  |  |  |  |  |  |  |  |  |  | WC & CF vs CF | 0.99  [-0.86, 2.85] | .29 |  |  |  |  |
|  | T2-T0 |  |  |  |  |  |  |  |  |  |  |  |  | WC & CF vs WC | -0.20  [-1.81, 1.40] | .80 |  |  |  |  |
|  | T3-T0 | 167 | -3.06 (13.28) | -3.12  [-4.72,-1.53] | 172 | -4.01 (14.75) | -3.33  [-4.91,-1.74] | 168 | -5.65 (12.78) | -4.79  [-6.40, -3.17] | 151 | -3.98 (12.92) | -4.99  [-6.62,-3.36] | CF vs TAU | -0.20  [-1.81, 1.40) | .80 |  |  |  |  |
|  | T3-T0 |  |  |  |  |  |  |  |  |  |  |  |  | WC vs TAU | -1.66  [-3.60, 0.28] | .09 |  |  |  |  |
|  | T3-T0 |  |  |  |  |  |  |  |  |  |  |  |  | WC vs CF | -1.46  [-3.96, 1.04] | .25 |  |  |  |  |
|  | T3-T0 |  |  |  |  |  |  |  |  |  |  |  |  | WC & CF vs TAU | -1.87  [-4.39, 0.66] | .15 |  |  |  |  |
|  | T3-T0 |  |  |  |  |  |  |  |  |  |  |  |  | WC & CF vs CF | -1.66  [-3.60, 0.28] | .09 |  |  |  |  |
|  | T3-T0 |  |  |  |  |  |  |  |  |  |  |  |  | WC & CF vs WC | -0.20  [-1.81, 1.40] | .80 |  |  |  |  |
|  | T4-T0 | 166 | -5.00 (14.63) | -4.37  [-5.96,-2.77] | 176 | -5.05 (13.65) | -4.57  [-6.15,-2.99] | 160 | -5.75 (14.98) | -5.00  [-6.62,-3.37] | 150 | -4.34 (15.95) | -5.20  [-6.84,-3.57] | CF vs TAU | -0.20  [-1.81, 1.40] | .80 |  |  |  |  |
|  | T4-T0 |  |  |  |  |  |  |  |  |  |  |  |  | WC vs TAU | -0.63  [-2.58, 1.31] | .52 |  |  |  |  |
|  | T4-T0 |  |  |  |  |  |  |  |  |  |  |  |  | WC vs CF | -0.43  [-2.93, 2.08] | 0.74 |  |  |  |  |
|  | T4-T0 |  |  |  |  |  |  |  |  |  |  |  |  | WC & CF vs TAU | -0.83  [-3.37, 1.70] | .52 |  |  |  |  |
|  | T4-T0 |  |  |  |  |  |  |  |  |  |  |  |  | WC & CF vs CF | -0.63  [-2.58, 1.31] | .52 |  |  |  |  |
|  | T4-T0 |  |  |  |  |  |  |  |  |  |  |  |  | WC & CF vs WC | -0.20  [-1.81, 1.40] | .80 |  |  |  |  |
| BSI - GSI | T0 | 276 | 0.36 (0.36) |  | 277 | 0.38 (0.37) |  | 280 | 0.39 (0.43) |  | 263 | 0.34 (0.32) |  |  |  |  | 0.26 | 0.01 | NA | 0.467 |
|  | T2-T0 | 185 | -0.04 (0.23) | -0.05  [-0.08, -0.02] | 202 | -0.08 (0.30) | -0.06  [-0.08,-0.03] | 184 | -0.03 (0.25) | -0.03  [-0.06, -0.00] | 186 | -0.03 (0.27) | -0.04  [-0.07,-0.01] | CF vs TAU | -0.00  [-0.03, 0.03] | .80 |  |  |  |  |
|  | T2-T0 |  |  |  |  |  |  |  |  |  |  |  |  | WC vs TAU | 0.02  [-0.02, 0.05] | .29 |  |  |  |  |
|  | T2-T0 |  |  |  |  |  |  |  |  |  |  |  |  | WC vs CF | 0.02  [-0.02, 0.07] | .34 |  |  |  |  |
|  | T2-T0 |  |  |  |  |  |  |  |  |  |  |  |  | WC & CF vs TAU | 0.01  [-0.03, 0.06] | .53 |  |  |  |  |
|  | T2-T0 |  |  |  |  |  |  |  |  |  |  |  |  | WC & CF vs CF | 0.02  [-0.02, 0.05] | .29 |  |  |  |  |
|  | T2-T0 |  |  |  |  |  |  |  |  |  |  |  |  | WC & CF vs WC | -0.00  [-0.03, 0.03] | .80 |  |  |  |  |
|  | T3-T0 | 167 | -0.06 (0.25) | -0.06  [-0.09, -0.03] | 172 | -0.08 (0.28) | -0.06  [-0.09,-0.03] | 168 | -0.11 (0.24) | -0.09  [-0.12, -0.06] | 151 | -0.08 (0.24) | -0.09  [-0.12,-0.06] | CF vs TAU | -0.00  [-0.03, 0.03] | .80 |  |  |  |  |
|  | T3-T0 |  |  |  |  |  |  |  |  |  |  |  |  | WC vs TAU | -0.03  [-0.07, 0.01] | .09 |  |  |  |  |
|  | T3-T0 |  |  |  |  |  |  |  |  |  |  |  |  | WC vs CF | -0.03  [-0.07, 0.02] | .25 |  |  |  |  |
|  | T3-T0 |  |  |  |  |  |  |  |  |  |  |  |  | WC & CF vs TAU | -0.04  [-0.08, 0.01] | .15 |  |  |  |  |
|  | T3-T0 |  |  |  |  |  |  |  |  |  |  |  |  | WC & CF vs CF | -0.03  [-0.07, 0.01] | .09 |  |  |  |  |
|  | T3-T0 |  |  |  |  |  |  |  |  |  |  |  |  | WC & CF vs WC | -0.00  [-0.03, 0.03] | .80 |  |  |  |  |
|  | T4-T0 | 166 | -0.09 (0.28) | -0.08  [-0.11, -0.05] | 176 | -0.10 (0.26) | -0.09  [-0.12,-0.06] | 160 | -0.11 (0.28) | -0.09  [-0.12, -0.06] | 150 | -0.08 (0.30) | -0.10  [-0.13,-0.07] | CF vs TAU | -0.00  [-0.03, 0.03] | .80 |  |  |  |  |
|  | T4-T0 |  |  |  |  |  |  |  |  |  |  |  |  | WC vs TAU | -0.01  [-0.05, 0.02] | .52 |  |  |  |  |
|  | T4-T0 |  |  |  |  |  |  |  |  |  |  |  |  | WC vs CF | -0.01  [-0.06, 0.04] | .74 |  |  |  |  |
|  | T4-T0 |  |  |  |  |  |  |  |  |  |  |  |  | WC & CF vs TAU | -0.02  [-0.06, 0.03] | .52 |  |  |  |  |
|  | T4-T0 |  |  |  |  |  |  |  |  |  |  |  |  | WC & CF vs CF | -0.01  [-0.05, 0.02] | .52 |  |  |  |  |
|  | T4-T0 |  |  |  |  |  |  |  |  |  |  |  |  | WC & CF vs WC | -0.00  [-0.03, 0.03] | .80 |  |  |  |  |
| BSI: Compulsiveness Mean | T0 | 285 | 0.59 (0.59) |  | 293 | 0.61 (0.59) |  | 295 | 0.63 (0.67) |  | 278 | 0.59 (0.55) |  |  |  |  | 0.48 | 0.02 | NA | 0.411 |
|  | T2-T0 | 193 | -0.04 (0.46) | -0.09  [-0.14,-0.04] | 216 | -0.13 (0.49) | -0.09  [-0.14,-0.05] | 197 | -0.07 (0.43) | -0.06  [-0.11,-0.01] | 206 | -0.05 (0.44) | -0.06  [-0.11,-0.02] | CF vs TAU | -0.01  [-0.06, 0.04] | .82 |  |  |  |  |
|  | T2-T0 |  |  |  |  |  |  |  |  |  |  |  |  | WC vs TAU | 0.03  [-0.03, 0.09] | .33 |  |  |  |  |
|  | T2-T0 |  |  |  |  |  |  |  |  |  |  |  |  | WC vs CF | 0.04  [-0.04, 0.11] | .37 |  |  |  |  |
|  | T2-T0 |  |  |  |  |  |  |  |  |  |  |  |  | WC & CF vs TAU | 0.02  [-0.05, 0.10] | .55 |  |  |  |  |
|  | T2-T0 |  |  |  |  |  |  |  |  |  |  |  |  | WC & CF vs CF | 0.03  [-0.03, 0.09] | .33 |  |  |  |  |
|  | T2-T0 |  |  |  |  |  |  |  |  |  |  |  |  | WC & CF vs WC | -0.01  [-0.06, 0.04] | .82 |  |  |  |  |
|  | T3-T0 | 172 | -0.08 (0.44) | -0.09  [-0.14, -0.03] | 189 | -0.10 (0.53) | -0.09  [-0.14,-0.04] | 176 | -0.17 (0.43) | -0.14  [-0.19,-0.09] | 165 | -0.11 (0.43) | -0.14  [-0.20,-0.09] | CF vs TAU | -0.01  [-0.06, 0.04] | .82 |  |  |  |  |
|  | T3-T0 |  |  |  |  |  |  |  |  |  |  |  |  | WC vs TAU | -0.05  [-0.12, 0.01] | .09 |  |  |  |  |
|  | T3-T0 |  |  |  |  |  |  |  |  |  |  |  |  | WC vs CF | -0.05  [-0.13, 0.03] | .24 |  |  |  |  |
|  | T3-T0 |  |  |  |  |  |  |  |  |  |  |  |  | WC & CF vs TAU | -0.06  [-0.14, 0.02] | .15 |  |  |  |  |
|  | T3-T0 |  |  |  |  |  |  |  |  |  |  |  |  | WC & CF vs CF | -0.05  [-0.12, 0.01] | .09 |  |  |  |  |
|  | T3-T0 |  |  |  |  |  |  |  |  |  |  |  |  | WC & CF vs WC | -0.01  [-0.06, 0.04] | .82 |  |  |  |  |
|  | T4-T0 | 170 | -0.13 (0.51) | -0.12  [-0.17, -0.07] | 187 | -0.14 (0.56) | -0.12  [-0.18,-0.07] | 169 | -0.16 (0.49) | -0.15  [-0.20,-0.10] | 161 | -0.15 (0.49) | -0.16  [-0.21,-0.10] | CF vs TAU | -0.01  [-0.06, 0.04] | .82 |  |  |  |  |
|  | T4-T0 |  |  |  |  |  |  |  |  |  |  |  |  | WC vs TAU | -0.03  [-0.09, 0.03] | .32 |  |  |  |  |
|  | T4-T0 |  |  |  |  |  |  |  |  |  |  |  |  | WC vs CF | -0.03  [-0.11, 0.05] | .52 |  |  |  |  |
|  | T4-T0 |  |  |  |  |  |  |  |  |  |  |  |  | WC & CF vs TAU | -0.04  [-0.12, 0.04] | 0.36 |  |  |  |  |
|  | T4-T0 |  |  |  |  |  |  |  |  |  |  |  |  | WC & CF vs CF | -0.03  [-0.09, 0.03] | 0.32 |  |  |  |  |
|  | T4-T0 |  |  |  |  |  |  |  |  |  |  |  |  | WC & CF vs WC | -0.01  [-0.06, 0.04) | 0.82 |  |  |  |  |
| BSI: Aggressiveness ans hostility Mean | T0 | 287 | 0.44 (0.48) |  | 292 | 0.52 (0.56) |  | 294 | 0.50 (0.55) |  | 278 | 0.42 (0.42) |  |  |  |  | 0.68 | 0.03 |  | 0.130 |
|  | T2-T0 | 194 | -0.04 (0.34) | -0.09  [-0.13,-0.05] | 216 | -0.17 (0.47) | -0.11  [-0.15,-0.07] | 196 | -0.09 (0.43) | -0.06  [-0.10,-0.02] | 206 | -0.04 (0.37) | -0.08  [-0.12,-0.04] | CF vs TAU | -0.02  [-0.06, 0.02] | .35 |  |  |  |  |
|  | T2-T0 |  |  |  |  |  |  |  |  |  |  |  |  | WC vs TAU | 0.03  [-0.02, 0.07] | .25 |  |  |  |  |
|  | T2-T0 |  |  |  |  |  |  |  |  |  |  |  |  | WC vs CF | 0.05  [-0.01, 0.11] | .14 |  |  |  |  |
|  | T2-T0 |  |  |  |  |  |  |  |  |  |  |  |  | WC & CF vs TAU | 0.01  [-0.05, 0.07] | .76 |  |  |  |  |
|  | T2-T0 |  |  |  |  |  |  |  |  |  |  |  |  | WC & CF vs CF | 0.03  [-0.02, 0.07] | .25 |  |  |  |  |
|  | T2-T0 |  |  |  |  |  |  |  |  |  |  |  |  | WC & CF vs WC | -0.02  [-0.06, 0.02] | .35 |  |  |  |  |
|  | T3-T0 | 173 | -0.09 (0.37) | -0.10  [-0.14,-0.06] | 189 | -0.14 (0.50) | -0.12  [-0.16,-0.08] | 176 | -0.17 (0.40) | -0.14  [-0.18,-0.10] | 166 | -0.12 (0.41) | -0.16  [-0.20,-0.11] | CF vs TAU | -0.02  [-0.06, 0.02] | .35 |  |  |  |  |
|  | T3-T0 |  |  |  |  |  |  |  |  |  |  |  |  | WC vs TAU | -0.04  [-0.09, 0.01] | .13 |  |  |  |  |
|  | T3-T0 |  |  |  |  |  |  |  |  |  |  |  |  | WC vs CF | -0.02  [-0.08, 0.04] | .52 |  |  |  |  |
|  | T3-T0 |  |  |  |  |  |  |  |  |  |  |  |  | WC & CF vs TAU | -0.06  [-0.12, 0.01] | .08 |  |  |  |  |
|  | T3-T0 |  |  |  |  |  |  |  |  |  |  |  |  | WC & CF vs CF | -0.04  [-0.09, 0.01] | .13 |  |  |  |  |
|  | T3-T0 |  |  |  |  |  |  |  |  |  |  |  |  | WC & CF vs WC | -0.02  [-0.06, 0.02] | .35 |  |  |  |  |
|  | T4-T0 | 170 | -0.14 (0.41) | -0.15  [-0.19,-0.11] | 190 | -0.20 (0.47) | -0.17  [-0.21,-0.13] | 167 | -0.20 (0.44) | -0.18  [-0.22,-0.13] | 160 | -0.17 (0.40) | -0.19  [-0.24,-0.15] | CF vs TAU | -0.02  [-0.06, 0.02] | .35 |  |  |  |  |
|  | T4-T0 |  |  |  |  |  |  |  |  |  |  |  |  | WC vs TAU | -0.03  [-0.08, 0.02] | .27 |  |  |  |  |
|  | T4-T0 |  |  |  |  |  |  |  |  |  |  |  |  | WC vs CF | -0.01  [-0.07, 0.05] | 0.76 |  |  |  |  |
|  | T4-T0 |  |  |  |  |  |  |  |  |  |  |  |  | WC & CF vs TAU | -0.05  [-0.11, 0.02] | .15 |  |  |  |  |
|  | T4-T0 |  |  |  |  |  |  |  |  |  |  |  |  | WC & CF vs CF | -0.03  [-0.08, 0.02] | .27 |  |  |  |  |
|  | T4-T0 |  |  |  |  |  |  |  |  |  |  |  |  | WC & CF vs WC | -0.02  [-0.06, 0.02] | .35 |  |  |  |  |
| PHQ-9 total value | T0 | 289 | 5.89 (4.52) |  | 296 | 6.07 (4.63) |  | 298 | 6.15 (4.57) |  | 280 | 5.74 (4.30) |  |  |  |  | 0.86 | 0.21 | NA | 0.267 |
|  | T2-T0 | 195 | -0.67 (3.70) | -0.74  [-1.08,-0.40] | 222 | -1.05 (3.56) | -0.88  [-1.21,-0.55] | 197 | -0.73 (3.01) | -0.77  [-1.10,-0.43] | 210 | -0.76 (3.31) | -0.91  [-1.24,-0.58] |  |  |  |  |  |  |  |
|  | T3-T0 | 175 | -0.62 (3.57) | -0.98  [-1.32,-0.64] | 192 | -1.41 (3.44) | -1.12  [-1.46,-0.79] | 177 | -1.31 (3.22) | -1.01  [-1.35,-0.67] | 168 | -0.83 (3.21) | -1.15  [-1.50,-0.81] |  |  |  |  |  |  |  |
|  | T4-T0 | 173 | -1.17 (4.10) | -1.27  [-1.61,-0.93] | 190 | -1.38 (3.75) | -1.41  [-1.75,-1.07] | 171 | -1.52 (3.48) | -1.30  [-1.64,-0.95] | 161 | -1.40 (3.60) | -1.44  [-1.79,-1.09] |  |  |  |  |  |  |  |
|  | All |  |  |  |  |  |  |  |  |  |  |  |  | CF vs TAU | -0.14  [-0.50, 0.21] | .43 |  |  |  |  |
|  | All |  |  |  |  |  |  |  |  |  |  |  |  | WC vs TAU | -0.03  [-0.38, 0.33] | .88 |  |  |  |  |
|  | All |  |  |  |  |  |  |  |  |  |  |  |  | WC vs CF | 0.11  [-0.38, 0.61] | .65 |  |  |  |  |
|  | All |  |  |  |  |  |  |  |  |  |  |  |  | WC & CF vs TAU | -0.17  [-0.68, 0.34] | .51 |  |  |  |  |
|  | All |  |  |  |  |  |  |  |  |  |  |  |  | WC & CF vs CF | -0.03  [-0.38, 0.33] | .88 |  |  |  |  |
|  | All |  |  |  |  |  |  |  |  |  |  |  |  | WC & CF vs WC | -0.14  [-0.50, 0.21] | .43 |  |  |  |  |
| ULQIE: total score | T0 | 263 | 2.58 (0.61) |  | 266 | 2.55 (0.61) |  | 266 | 2.57 (0.55) |  | 252 | 2.56 (0.55) |  |  |  |  | 0.74 | 0.52 | NA | 0.072 |
|  | T2-T0 | 170 | 0.03 (0.40) | 0.06  [0.00, 0.11] | 177 | 0.12 (0.44) | 0.06  [0.01, 0.12] | 168 | 0.08 (0.45) | 0.06  [0.00, 0.11] | 175 | 0.03 (0.46) | 0.06  [0.01, 0.12] |  |  |  |  |  |  |  |
|  | T3-T0 | 151 | 0.08 (0.43) | 0.12  [0.06, 0.17] | 158 | 0.15 (0.44) | 0.12  [0.07, 0.18] | 152 | 0.15 (0.41) | 0.12  [0.06, 0.17] | 143 | 0.11 (0.43) | 0.12  [0.07, 0.18] |  |  |  |  |  |  |  |
|  | T4-T0 | 153 | 0.12 (0.48) | 0.14  [0.08, 0.19] | 151 | 0.16 (0.42) | 0.14  [0.09, 0.20] | 146 | 0.18 (0.46) | 0.14  [0.08, 0.19] | 142 | 0.13 (0.46) | 0.14  [0.09, 0.20] |  |  |  |  |  |  |  |
|  | All |  |  |  |  |  |  |  |  |  |  |  |  | CF vs TAU | 0.01  [-0.05, 0.06] | .82 |  |  |  |  |
|  | All |  |  |  |  |  |  |  |  |  |  |  |  | WC vs TAU | 0.00  [-0.05, 0.05] | .99 |  |  |  |  |
|  | All |  |  |  |  |  |  |  |  |  |  |  |  | WC vs CF | -0.01  [-0.08, 0.07] | .88 |  |  |  |  |
|  | All |  |  |  |  |  |  |  |  |  |  |  |  | WC & CF vs TAU | 0.01  [-0.07, 0.08] | .87 |  |  |  |  |
|  | All |  |  |  |  |  |  |  |  |  |  |  |  | WC & CF vs CF | 0.00  [-0.05, 0.05] | .99 |  |  |  |  |
|  | All |  |  |  |  |  |  |  |  |  |  |  |  | WC & CF vs WC | 0.01  [-0.05, 0.06] | .82 |  |  |  |  |

**Table S2** *CARE-FAM-NET: Only Distressed Parents*

|  | | **TAU** | | | **CF** | | **WC** | | | | **CF&WC** | | | | |  | | | | **Interaction p-value** | | | |
| --- | --- | --- | --- | --- | --- | --- | --- | --- | --- | --- | --- | --- | --- | --- | --- | --- | --- | --- | --- | --- | --- | --- | --- |
| **Variable** | **Time** | ***n*** | ***M* (*SD*)** | **adj. *M* [95%CI]** | ***n*** | ***M* (*SD*)** | **adj. *M* [95%CI]** | ***n*** | ***M* (*SD*)** | **adj. *M* [95%CI]** | | ***n*** | ***M* (*SD*)** | **adj. *M* [95%CI]** | **Comparison** | | **Diff [95%CI]** | ***p*** | **CF-time** | | **WC-time** | **CF-WC-time** | **CF-WC** |
| BSI - TS | T0 | 67 | 44.37 (21.22) |  | 76 | 46.12 (17.98) |  | 57 | 53.93 (28.11) |  | | 52 | 43.54 (17.49) |  |  | |  |  | 0.46 | | 0.02 | NA | 0.814 |
|  | T2-T0 | 27 | -14.67 (17.14) | -13.71  [-20.9,-6.56] | 46 | -12.91 (26.84) | -13.99  [-20.5,-7.51] | 23 | -8.13 (29.92) | -5.12  [-13.07, 2.82] | | 17 | -11.47 (24.37) | -5.41  [-13.79, 2.97] | CF vs TAU | | -0.29  [-7.98, 7.40] | .94 |  | |  |  |  |
|  | T2-T0 |  |  |  |  |  |  |  |  |  | |  |  |  | WC vs TAU | | 8.58  [-0.50,17.66] | .06 |  | |  |  |  |
|  | T2-T0 |  |  |  |  |  |  |  |  |  | |  |  |  | WC vs CF | | 8.87  [-2.30,20.05] | .12 |  | |  |  |  |
|  | T2-T0 |  |  |  |  |  |  |  |  |  | |  |  |  | WC & CF vs TAU | | 8.30  [-4.28,20.87] | .19 |  | |  |  |  |
|  | T2-T0 |  |  |  |  |  |  |  |  |  | |  |  |  | WC & CF vs CF | | 8.58  [-0.50,17.66] | .06 |  | |  |  |  |
|  | T2-T0 |  |  |  |  |  |  |  |  |  | |  |  |  | WC & CF vs WC | | -0.29  [-7.98, 7.40] | .94 |  | |  |  |  |
|  | T3-T0 | 26 | -12.46 (22.76) | -11.42  [-18.6,-4.20] | 40 | -9.38 (24.45) | -11.70  [-18.35,-5.06)] | 21 | -17.48 (26.73) | -15.16  [-23.36, -6.96] | | 14 | -19.07 (24.53) | -15.45  [-24.14, -6.76] | CF vs TAU | | -0.29  [-7.98, 7.40] | .94 |  | |  |  |  |
|  | T3-T0 |  |  |  |  |  |  |  |  |  | |  |  |  | WC vs TAU | | -3.74  [-13.1, 5.65] | .43 |  | |  |  |  |
|  | T3-T0 |  |  |  |  |  |  |  |  |  | |  |  |  | WC vs CF | | -3.46  [-14.89,7.97] | .55 |  | |  |  |  |
|  | T3-T0 |  |  |  |  |  |  |  |  |  | |  |  |  | WC & CF vs TAU | | -4.03  [-16.83,8.77] | .54 |  | |  |  |  |
|  | T3-T0 |  |  |  |  |  |  |  |  |  | |  |  |  | WC & CF vs CF | | -3.74  [-13.14,5.65] | .43 |  | |  |  |  |
|  | T3-T0 |  |  |  |  |  |  |  |  |  | |  |  |  | WC & CF vs WC | | -0.29  [-7.98, 7.40] | .94 |  | |  |  |  |
|  | T4-T0 | 30 | -20.43 (18.98) | -15.41  [-22.6, -8.26] | 39 | -12.41 (21.32) | -15.70  [-22.35, -9.05] | 23 | -17.65 (32.69) | -16.53  [-24.48, -8.58] | | 17 | -20.71 (32.78) | -16.82  [-25.24, -8.40] | CF vs TAU | | -0.29  [-7.98, 7.40] | .94 |  | |  |  |  |
|  | T4-T0 |  |  |  |  |  |  |  |  |  | |  |  |  | WC vs TAU | | -1.12  [-10.25,8.02] | .81 |  | |  |  |  |
|  | T4-T0 |  |  |  |  |  |  |  |  |  | |  |  |  | WC vs CF | | -0.83  [-12.1, 10.46] | .88 |  | |  |  |  |
|  | T4-T0 |  |  |  |  |  |  |  |  |  | |  |  |  | WC & CF vs TAU | | -1.40  [-13.95, 11.2] | .83 |  | |  |  |  |
|  | T4-T0 |  |  |  |  |  |  |  |  |  | |  |  |  | WC & CF vs CF | | -1.12  [-10.25, 8.02] | .81 |  | |  |  |  |
|  | T4-T0 |  |  |  |  |  |  |  |  |  | |  |  |  | WC & CF vs WC | | -0.29  [-7.98, 7.40] | .94 |  | |  |  |  |
| BSI - GSI | T0 | 67 | 0.84 (0.40) |  | 76 | 0.87 (0.34) |  | 57 | 1.02 (0.53) |  | | 52 | 0.82 (0.33) |  |  | |  |  | 0.46 | | 0.02 | NA | 0.814 |
|  | T2-T0 | 27 | -0.28 (0.32) | -0.26  [-0.39,-0.12] | 46 | -0.24 (0.51) | -0.26  [-0.39,-0.14] | 23 | -0.15 (0.56) | -0.10  [-0.25, 0.05] | | 17 | -0.22 (0.46) | -0.10  [-0.26, 0.06] | CF vs TAU | | -0.01  [-0.15, 0.14] | .94 |  | |  |  |  |
|  | T2-T0 |  |  |  |  |  |  |  |  |  | |  |  |  | WC vs TAU | | 0.16  [-0.01, 0.33] | .06 |  | |  |  |  |
|  | T2-T0 |  |  |  |  |  |  |  |  |  | |  |  |  | WC vs CF | | 0.17  [-0.04, 0.38] | .12 |  | |  |  |  |
|  | T2-T0 |  |  |  |  |  |  |  |  |  | |  |  |  | WC & CF vs TAU | | 0.16  [-0.08, 0.39] | .19 |  | |  |  |  |
|  | T2-T0 |  |  |  |  |  |  |  |  |  | |  |  |  | WC & CF vs CF | | 0.16  [-0.01, 0.33] | .06 |  | |  |  |  |
|  | T2-T0 |  |  |  |  |  |  |  |  |  | |  |  |  | WC & CF vs WC | | -0.01  [-0.15, 0.14] | .94 |  | |  |  |  |
|  | T3-T0 | 26 | -0.24 (0.43) | -0.22  [-0.35,-0.08] | 40 | -0.18 (0.46) | -0.22  [-0.35,-0.10] | 21 | -0.33 (0.50) | -0.29  [-0.44,-0.13] | | 14 | -0.36 (0.46) | -0.29  [-0.46, -0.13] | CF vs TAU | | -0.01  [-0.15, 0.14] | .94 |  | |  |  |  |
|  | T3-T0 |  |  |  |  |  |  |  |  |  | |  |  |  | WC vs TAU | | -0.07  [-0.25, 0.11] | .43 |  | |  |  |  |
|  | T3-T0 |  |  |  |  |  |  |  |  |  | |  |  |  | WC vs CF | | -0.07  [-0.28, 0.15] | .55 |  | |  |  |  |
|  | T3-T0 |  |  |  |  |  |  |  |  |  | |  |  |  | WC & CF vs TAU | | -0.08  [-0.32, 0.17] | .54 |  | |  |  |  |
|  | T3-T0 |  |  |  |  |  |  |  |  |  | |  |  |  | WC & CF vs CF | | -0.07  [-0.25, 0.11] | .43 |  | |  |  |  |
|  | T3-T0 |  |  |  |  |  |  |  |  |  | |  |  |  | WC & CF vs WC | | -0.01  [-0.15, 0.14] | .94 |  | |  |  |  |
|  | T4-T0 | 30 | -0.39 (0.36) | -0.29  [-0.43,-0.16] | 39 | -0.23 (0.40) | -0.30  [-0.42,-0.17] | 23 | -0.33 (0.62) | -0.31  [-0.46,-0.16] | | 17 | -0.39 (0.62) | -0.32  [-0.48, -0.16] | CF vs TAU | | -0.01  [-0.15, 0.14] | .94 |  | |  |  |  |
|  | T4-T0 |  |  |  |  |  |  |  |  |  | |  |  |  | WC vs TAU | | -0.02  [-0.19, 0.15] | .81 |  | |  |  |  |
|  | T4-T0 |  |  |  |  |  |  |  |  |  | |  |  |  | WC vs CF | | -0.02  [-0.23, 0.20] | .88 |  | |  |  |  |
|  | T4-T0 |  |  |  |  |  |  |  |  |  | |  |  |  | WC & CF vs TAU | | -0.03  [-0.26, 0.21] | .83 |  | |  |  |  |
|  | T4-T0 |  |  |  |  |  |  |  |  |  | |  |  |  | WC & CF vs CF | | -0.02  [-0.19, 0.15] | .81 |  | |  |  |  |
|  | T4-T0 |  |  |  |  |  |  |  |  |  | |  |  |  | WC & CF vs WC | | -0.01  [-0.15, 0.14] | .94 |  | |  |  |  |
